# Effects of returning peach branch waste to fields on soil carbon cycle mediated by soil microbial communities

**DOI:** 10.3389/fmicb.2024.1406661

**Published:** 2024-06-18

**Authors:** Chenyu Liu, Zhiling Liu, Bofei Cui, Haiqing Yang, Chengda Gao, Mingming Chang, Yueping Liu

**Affiliations:** ^1^College of Bioscience and Resource Environment, Beijing University of Agriculture, Beijing, China; ^2^Fruit Industry Serve Center of Pinggu District, Beijing, China; ^3^College of Humanities and Urban-Rural Development, Beijing University of Agriculture, Beijing, China; ^4^Key Laboratory for Northern Urban Agriculture Ministry of Agriculture and Rural Affairs, Beijing University of Agriculture, Beijing, China

**Keywords:** peach branch waste, soil chemical property, soil organic carbon, CO_2_ fluxes, soil microbial community

## Abstract

In recent years, the rise in greenhouse gas emissions from agriculture has worsened climate change. Efficiently utilizing agricultural waste can significantly mitigate these effects. This study investigated the ecological benefits of returning peach branch waste to fields (RPBF) through three innovative strategies: (1) application of peach branch organic fertilizer (OF), (2) mushroom cultivation using peach branches as a substrate (MC), and (3) surface mulching with peach branches (SM). Conducted within a peach orchard ecosystem, our research aimed to assess these resource utilization strategies’ effects on soil properties, microbial community, and carbon cycle, thereby contributing to sustainable agricultural practices. Our findings indicated that all RPBF treatments enhance soil nutrient content, enriching beneficial microorganisms, such as *Humicola*, Rhizobiales, and *Bacillus*. Moreover, soil AP and AK were observed to regulate the soil carbon cycle by altering the compositions and functions of microbial communities. Notably, OF and MC treatments were found to boost autotrophic microorganism abundance, thereby augmenting the potential for soil carbon sequestration and emission reduction. Interestingly, in peach orchard soil, fungal communities were found to contribute more greatly to SOC content than bacterial communities. However, SM treatment resulted in an increase in the presence of bacterial communities, thereby enhancing carbon emissions. Overall, this study illustrated the fundamental pathways by which RPBF treatment affects the soil carbon cycle, providing novel insights into the rational resource utilization of peach branch waste and the advancement of ecological agriculture.

## 1 Introduction

Over the past century, with the continuous increase in carbon dioxide (CO_2_) emissions and global temperatures, more unstable and extreme weather will occur, thus the issue of climate change has attracted global attention (Jansson and Hofmockel, 2020; Zhang X. et al., 2022). Agriculture is closely related to greenhouse gas (GHG) emissions. Although global agricultural GHG emissions continued to increase, agriculture had enormous potential in reducing GHG emissions (Yu et al., 2020; Khatri-Chhetri et al., 2022). Notably, burning crop residues and synthetic fertilizers are important sources of agricultural GHG emissions (FAO, 2023). However, crop residues return instead of burning them have been shown to increase soil carbon, nitrogen, and phosphorus content, which helped to mitigate climate change (Liu J. et al., 2023), while excessive use of chemical fertilizers not only led to greenhouse gas emissions but also exacerbated soil acidification (Haq et al., 2020; Zhang Y. et al., 2022). Therefore, the rational resource utilization of agricultural waste and the reduction of fertilizer application are particularly important. Agricultural waste can be effectively utilized as surface mulch, fertilizers, and as a growing medium for mushrooms (Koul et al., 2022). These methods of managing agricultural waste could contribute to sustainable agriculture and the environment. For instance, a forage maize field study shows that straw mulching reduced soil CO_2_ fluxes and increased yield compared to no mulching (Fan et al., 2019). Moreover, mushroom cultivation and fertilization could increase soil organic matter (SOM) content and enhance soil quality (Wu et al., 2020; Hu et al., 2021).

Peach [*Prunus persica* (L.) Batsch], a member of the Rosaceae family, is a globally significant fruit crop. It is renowned for its considerable economic value and nutritional benefits. China, as the origin of peaches, boasts a long history of cultivation and a wide range of planting areas (Liu et al., 2015; Cai et al., 2023). In 2022, China’s peach production accounted for 63.81% of the world’s peach production. Between 2002 and 2022, the peach area harvested in China increased from approximately 0.55 million hectares (Mh) to 0.87 Mh. Concurrently, peach production increased from approximately 5.29 million tons (Mt) to 16.82 Mt (FAO, 2023). Over the last two decades, the peach industry has seen rapid development, resulting in an increased production of peach branch waste, which is a form of agricultural waste. Traditionally, these wastes were disposed of through burning or stacking in fields, contributing significantly to environmental pollution (Wei et al., 2023). Consequently, the efficient and sustainable utilization of peach branch waste has become a critical issue. Recent studies have shown that sawdust, derived from crushed peach branch waste, can be effectively utilized as a substrate for the cultivation of oyster mushrooms (Guo et al., 2021; Yang et al., 2022). Additionally, our previous research demonstrated that peach branch organic fertilizer (PBOF) not only improved peach yield but also increased soil nutrients (Liu C. et al., 2023). However, the impact of returning peach branch waste to fields (RPBF) on climate change mitigation is less explored and deserves further investigation.

Soil microorganisms play essential roles in ecological functions, such as climate regulation, nutrient cycling, plant growth promotion, disease control, and pollutant degradation (French et al., 2021; Frąc et al., 2022; Hartmann and Six, 2023). In terms of climate regulation, soil microorganisms mitigate or accelerate climate change by influencing the carbon cycle, specifically through the promotion of organic carbon sequestration or CO_2_ emissions (Singh et al., 2010; Naylor et al., 2020; Jiang et al., 2022). Soil organic carbon (SOC) not only maintains ecosystem health but also serves as an energy source for plant growth and soil organisms. Therefore, reducing SOC loss and sequestering CO_2_ as SOC in soil is particularly crucial (Tao et al., 2023; Wu et al., 2024). Microorganisms not only reduce SOC through mineralization to CO_2_ but also accumulate microbial biomass and byproducts through biosynthetic metabolism (Tao et al., 2023). This microbial biomass eventually transforms into necromass. Both necromass and by-products contribute to SOC formation (Liang et al., 2017, 2019; Tao et al., 2023). In addition, autotrophic microorganisms can also immobilize CO_2_ into organic matter, contributing to SOC (Liu et al., 2018; Jiang et al., 2022; Wu et al., 2024). Furthermore, many studies have shown that agricultural management strategies changed the diversity, composition, and function of microbial communities, thereby affecting the accumulation of SOC and CO_2_ emissions (Pratibha et al., 2023; Singh et al., 2023; Tian et al., 2024). For example, straw returning changed microbial community composition (such as the abundance of Proteobacteria, Mortierellomycota, and Glomeromycota) to increase SOC content (Cong et al., 2024). Thus, the role of soil microorganisms should not be overlooked when assessing the impact of RPBF on SOC content and CO_2_ emission.

In this study, we aimed to reveal the contributions of RPBF to carbon sequestration and emission reduction, thereby offering potential agricultural management strategies for mitigating climate change. Specifically, our objectives were to (1) analyze the effects of RPBF on soil properties and the microbial community; (2) investigate the influence of RPBF on SOC and CO_2_ fluxes; (3) explore the potential correlation between soil properties, microbial community, and variations in SOC and CO_2_ fluxes. There were two main hypotheses: (1) RPBF may improve soil properties and have an impact on soil microbial community composition and function; and (2) RPBF may contribute to SOC accumulation and influence CO_2_ emissions, regulated by the microbial community. This study is expected to provide novel insights into the role of RPBF in enhancing soil carbon sequestration and reducing emissions, contributing to the advancement of green agricultural practices and environmental sustainability.

## 2 Materials and methods

### 2.1 Study site and experimental design

Our study was carried out in 2021 at Yindong Village, Liujiadian Town, Pinggu District, Beijing (40°14′N, 117°2′E). The annual average temperature in this region was 11.7°C, with an average precipitation of 630 mm and an annual average sunshine duration of 2,519 h. The soil type was classified as Haplustalf (United States Department of Agriculture). In this peach orchard, the planting distance between peach trees was 2 m × 4 m. Detailed chemical properties of the peach orchard soil were shown in Table S1.

This experiment site consisted of four blocks, each block subjected to a distinct treatment: (1) CK, no treatment; (2) OF, application of organic fertilizer; (3) MC, mushroom cultivation; (4) SM, surface mulching. Each treatment had four replicates. The three RPBF treatments were all applied around the peach trees in their blocks, and the application time was in the autumn of 2021. Other practices in the peach orchard were carried out according to normal field management methods.

The organic fertilizer, mushroom cultivation substrate, and mulching material all use peach branch waste as the main raw material. The organic fertilizer was purchased from Beijing Dahua Fertilizer Industry Co., Ltd., and applied using furrow fertilization, with an application rate of 3,500 kg/667 m^2^ and a fertilization depth of approximately 15 cm. Peach branches and corn straws were crushed into 3–5 cm pieces and mixed in a ratio of 7:3. This mixture was then composted for 7–15 days to create the cultivation substrate and then distributed over the block with a thickness of approximately 25 cm, inoculating wine cap mushrooms (*Stropharia rugosoannulata*). After the mushroom harvest, the mushroom cultivation substrate was not removed. The surface mulch was made by crushing peach branches into 3–5 cm pieces composting for 7–15 days and then spreading it on the block with a thickness of about 25 cm. The chemical properties of these materials were also shown in Table S1.

### 2.2 Determination of CO_2_ fluxes and temperature

At the outset of the experiment, gas sampling chambers (31 cm in diameter) were strategically placed between the peach trees in four blocks, with a distance of 2 m between each gas sampling chamber. In the summer (May, June, July) and autumn (August, September, October) of 2022, measurements of CO_2_ fluxes and temperature were conducted respectively, and taken in the morning of the day, with four replicates of each treatment. The initial concentration of CO_2_ within the chambers was determined using the GXH-3010H handheld infrared CO_2_ analyzer (Beijing Hua Yun Analytical Instrument Research Institute Co., Ltd.), followed by a second measurement 3 min later. Moreover, the temperature inside the chambers was concurrently recorded using a thermometer.

### 2.3 Soil sampling and analysis of soil chemical properties

In November 2022, we collected soil samples from each block at a depth of 0–20 cm. These soil samples were passed through a 2 mm sieve to remove visible plant roots and stones. Subsequently, each soil sample was divided into three parts. One part was stored at −80°C for DNA extraction, while the other part was air-dried and used for the analysis of soil pH, total carbon (TC), soil organic carbon (SOC), available nitrogen (AN), available phosphorus (AP), and available potassium (AK). The last part was stored at −20°C to measure microbial biomass carbon (MBC). We applied a pH meter to measure the soil pH with a soil to water ratio of 1:2.5 (w/v). TC and SOC were analyzed by dry combustion (ISO 10694: 1995). AN was extracted with sodium chloride and determined by zinc-sulfuric acid ferrous distillation method. AP was determined by the molybdenum blue spectrophotometry method. AK was extracted with ammonium acetate and determined by flame photometry. MBC was evaluated using the chloroform fumigation extraction method (Vance et al., 1987).

### 2.4 DNA extraction, amplification and sequencing

Soil genomic DNA was extracted from 0.25 g of each soil sample using E.Z.N.A. Soil DNA Kit (Omega Bio-tek, Inc., USA) following the manufacturer’s instructions. The concentration and quality of the genomic DNA were assessed using a NanoDrop 2000 spectrophotometer (Thermo Scientific Inc., USA). The V3-V4 region of the bacterial 16S rRNA gene was amplified using primers 338F (ACTCCTACGGGAGGCAGCA) and 806R (GGACTACHVGGGTWTCTAAT), while the ITS region of the fungal DNA gene using primers ITS5F (GGAAGTAAAAGTCGTAACAAGG) and ITS2R (GCTGCGTTCTTCATCGATGC) (Bellemain et al., 2010; Nossa et al., 2010). The PCR products were purified using the Agencourt AMPure XP Kit (Beckman Coulter, Inc., USA). Deep sequencing was performed on the Illumina Novaseq PE250 (Illumina, USA) platform by Beijing Allwegene Technology Co., Ltd., (China).

### 2.5 Data analysis

Use Pear (version 0.9.6) software to quality control and splice raw data (Zhang et al., 2014). VSEARCH (version 2.7.1) software was used to remove the chimeric sequence by the UCHIME method (Edgar et al., 2011; Rognes et al., 2016). The qualified sequences were clustered into operational taxonomic units (OTUs) at a similarity threshold of 97% using the UPARSE algorithm of VSEARCH (version 2.7.1) software (Edgar, 2013). The OTU representative sequences of bacteria and fungi were annotated using the Silva (version 138) and the Unite (version 8.2) databases, respectively.

The alpha diversity indices, including Chao1 and Shannon, were calculated using the vegan package in R (version 4.3.1). The principal coordinates analysis (PCoA), based on Bray–Curtis distance, was used to assess the variation of bacterial and fungal communities. Permutational multivariate analyses of variance (PERMANOVA) were performed to evaluate the effects of RPBF treatments on bacterial and fungal communities using the Adonis function. Multilevel pairwise comparisons were conducted using the pairwiseAdonis package (Cuartero et al., 2022). The linear discriminant analysis (LDA) effect size (LEfSe) was realized on the website http://huttenhower.sph.harvard.edu/galaxy/. The biomarkers of soil bacteria and fungi in each treatment were identified, and all microbial taxa had LDA scores >4 (Segata et al., 2011). We used the FAPROTAX database to predict the functions of bacterial biogeochemical cycles (Louca et al., 2016), while the FUNGuild database was used to obtain fungal functional guild annotation (Nguyen et al., 2016). To reveal the effects of RPBF on co-occurrence patterns of bacterial and fungal OTUs in all soil samples, network analysis was conducted. The OTUs considered for this analysis were those with a relative abundance greater than 0.05% and present in more than 75% of all soil samples, and the Hmisc package within R (version 4.3.1) was used to calculate Spearman’s correlation matrix (Wang Z. et al., 2022; Tang et al., 2023). The network was constructed using a strong Spearman’s correlation coefficient with an absolute *r* > 0.80 and *p* < 0.01, and then the topological characteristics of the networks were calculated using the igraph package in R (version 4.3.1), and Gephi (version 0.10.1) was used for visualization. In addition, Spearman correlation analysis of soil microorganisms and environmental factors used the psych package, and the Mantel test was performed using the linkET package in R (version 4.3.1).

Structural equation modeling (SEM) was conducted using AMOS software (SPSS AMOS 27.0.0). A non-significant chi-square value (*p* > 0.05), goodness of fit index (>0.9), comparative fit index (>0.9), and the root mean square error of approximation (<0.08) reflected a good fitting of the SEMs. Significant differences in average temperature, average CO_2_ fluxes, soil chemical properties, α-diversity index, composition and function of soil microbial community across different treatments were analyzed using one-way analysis of variance (ANOVA) and Fisher’s LSD test (*p* < 0.05) on OriginPro 2023b (OriginLab Corp., United States).

## 3 Results

### 3.1 Effects of RPBF on soil chemical properties

In this study, we observed that each RPBF treatment significantly increased (*p* < 0.05) soil pH (Supplementary Table S2). The OF, MC, and SM treatments resulted in an increase in TC by 92, 222, and 338%, respectively. These treatments also augmented SOC by 50, 148, and 333%, correspondingly. Additionally, the SM treatment showed a significant enhancement in both MBC and AK, compared to CK (*p* < 0.05). Furthermore, The MC and SM treatments significantly improved soil AP (*p* < 0.05). Although all RPBF treatments exhibited an increase in soil AN, there was no significant difference (*p* > 0.05).

### 3.2 Effects of RPBF on temperature and CO_2_ fluxes

**Figure 1:**
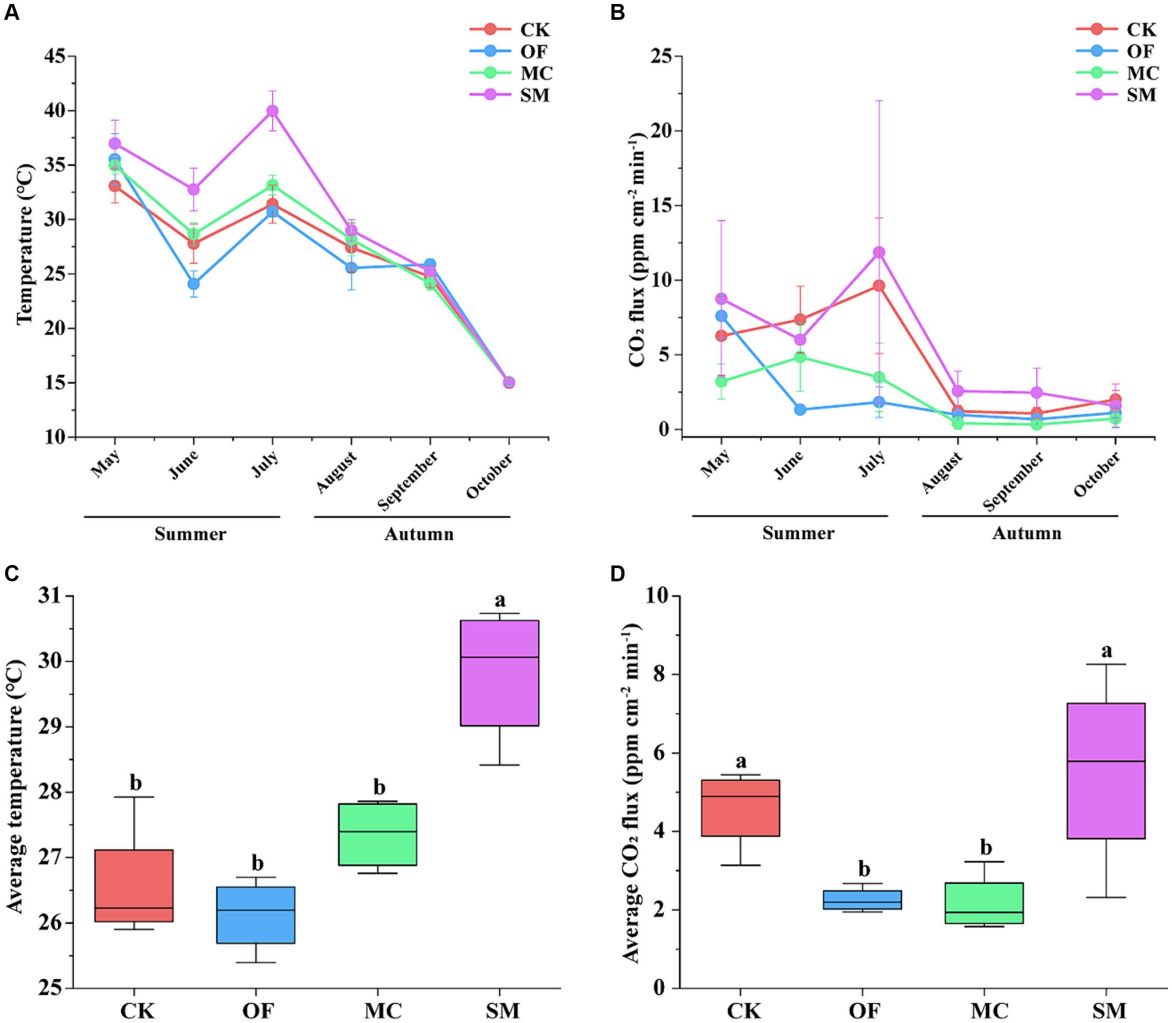
Changes in temperature and CO_2_ fluxes in different seasons under RPBF treatments. The line diagram shows the fluctuation of temperature **(A)** and CO_2_ fluxes **(B)**. Effects of different treatments on average temperature **(C)** and average CO_2_ fluxes **(D)**. CK, no treatment; OF, application of organic fertilizer; MC, mushroom cultivation; SM, surface mulching. The different letters in the figure indicate a significant difference at *p* < 0.05.

In all treatments, temperature and CO_2_ fluxes varied with seasonal changes, showing a fluctuating downward trend from summer to autumn (Figures 1A,B). In summer, the temperature and CO_2_ fluxes were the highest in the SM treatment. Furthermore, the CO_2_ fluxes under the OF and MC treatments were lower than those in the CK treatment. However, in autumn, there were no obvious variations among the four treatments. Compared to CK, the SM treatment increased the average temperature by 3.25°C and the average CO_2_ fluxes by 0.95 ppm cm^−2^ min^−1^ (Figures 1C,D). However, the OF and MC treatments significantly decreased average CO_2_ fluxes (*p* < 0.05), but there was no significant difference in temperature (*p* > 0.05).

### 3.3 Effects of RPBF on microbial diversity

**Figure 2:**
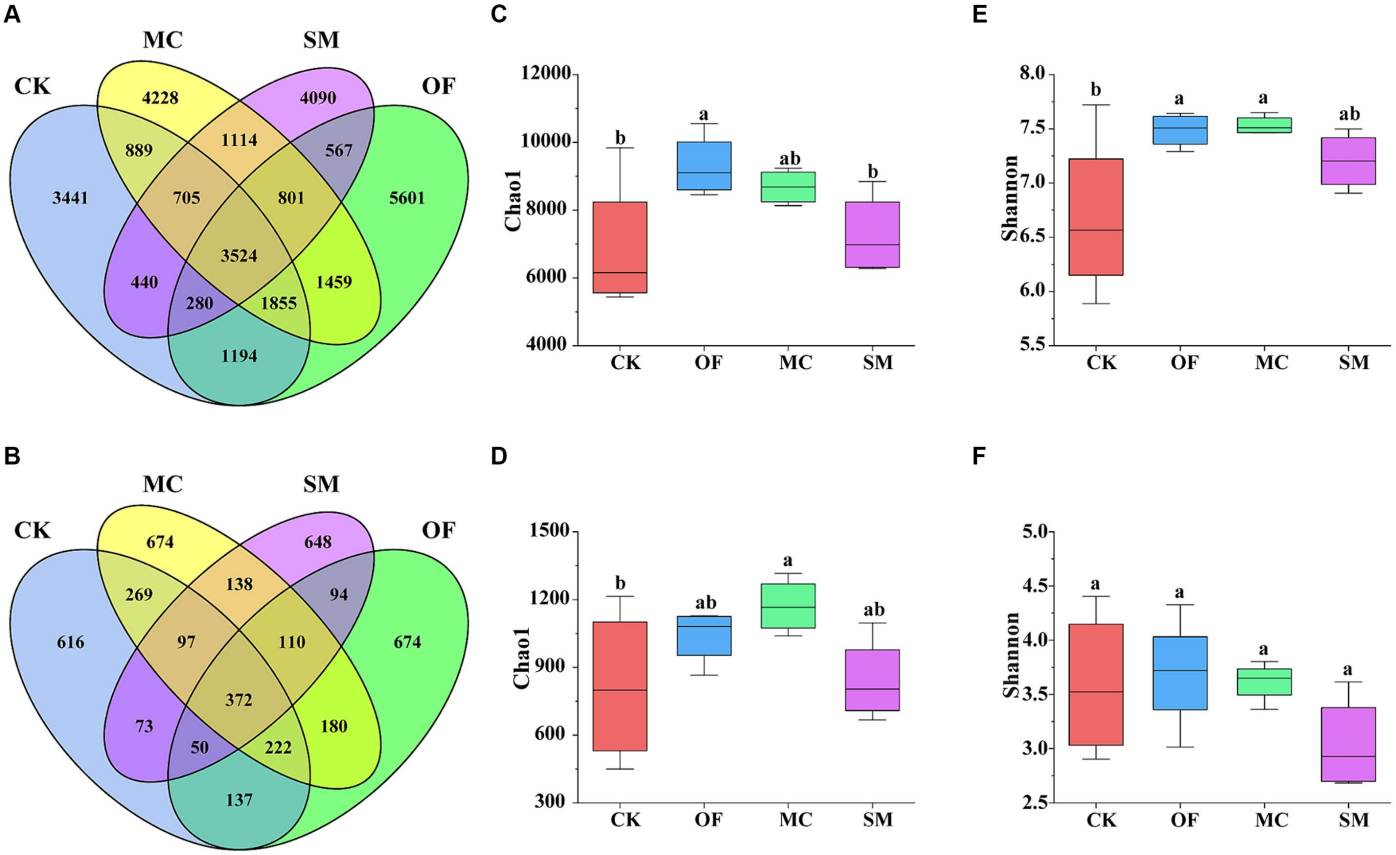
The differences in microbial diversity under RPBF treatments. Venn diagram showing the common and unique OTUs of bacteria **(A)** and fungi **(B)**. Effects of different treatments on the alpha diversity of bacteria **(C,E)** and fungi **(D,F)**. CK, no treatment; OF, application of organic fertilizer; MC, mushroom cultivation; SM, surface mulching. The different letters in the figure indicate a significant difference at *p* < 0.05.

We analyzed the impact of different treatments on the diversity of bacteria and fungi. The number of common bacterial OTUs was 3,524. Compared to the CK treatment, the OF, MC, and SM treatments all increased the number of unique bacterial OTUs, with increases of 2,160, 787, and 649, respectively (Figure 2A). The OF treatment significantly led to a significant increase in the bacterial Chao1 index (*p* < 0.05; Figure 2C). Moreover, the OF and MC treatments significantly augmented the bacterial Shannon index (*p* < 0.05; Figure 2E). For fungi, the number of common fungal OTUs was 372. Compared to CK, the number of unique fungal OTUs increased by 58, 32, and 58 for OF, MC, and SM treatments, respectively (Figure 2B). Additionally, the MC treatment was particularly notable for significantly increased the fungal Chao1 index (*p* < 0.05; Figure 2D). However, there were no significant differences in fungal Shannon index under different treatments (*p* > 0.05; Figure 2F).

**Figure 3:**
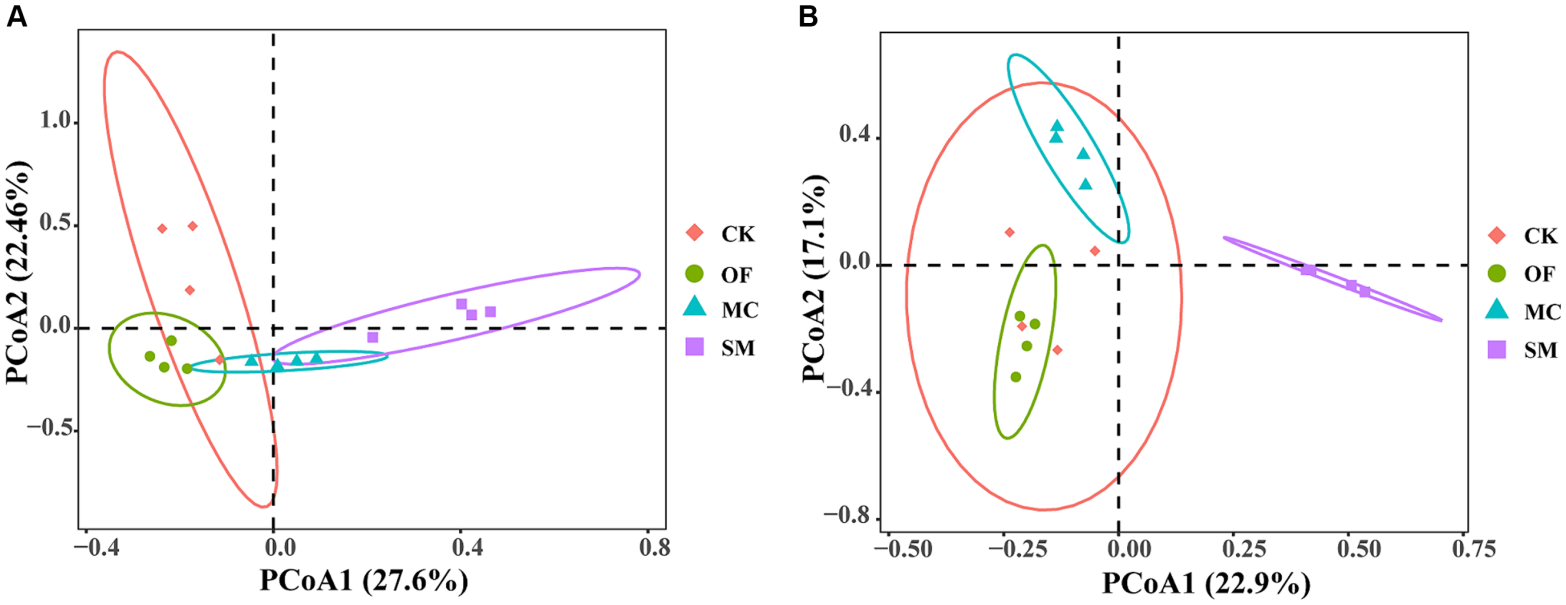
Principal coordinate analysis (PCoA) of bacteria **(A)** and fungi **(B)** under different treatments. CK, no treatment; OF, application of organic fertilizer; MC, mushroom cultivation; SM, surface mulching.

Based on principal coordinate analysis (PCoA), we found that the bacterial and fungal communities from different treatments clustered together separately (Figure 3). Furthermore, different treatments explained 51.5% of total variations in bacterial community compositions (PERMANOVA: *R*^2^ = 0.515, *p* = 0.001) and 48.6% of total variations in fungal community compositions (PERMANOVA: *R*^2^ = 0.486, *p* = 0.001). The pairwise adonis test results further revealed that there was no significant difference in the bacterial community composition between the CK and OF treatments (*R*^2^ = 0.328, *p* = 0.064), while there were significant differences in the bacterial community composition between the CK treatment and both the MC (*R*^2^ = 0.356, *p* = 0.034) and SM (*R*^2^ = 0.392, *p* = 0.035) treatments (Supplementary Table S3). Additionally, fungal community compositions differed significantly between the CK treatment and all RPBF treatments (*p* < 0.05). Overall, there were significant differences in both bacterial and fungal community compositions across the three RPBF treatments (*p* < 0.05).

### 3.4 Effects of RPBF on microbial community composition

**Figure 4:**
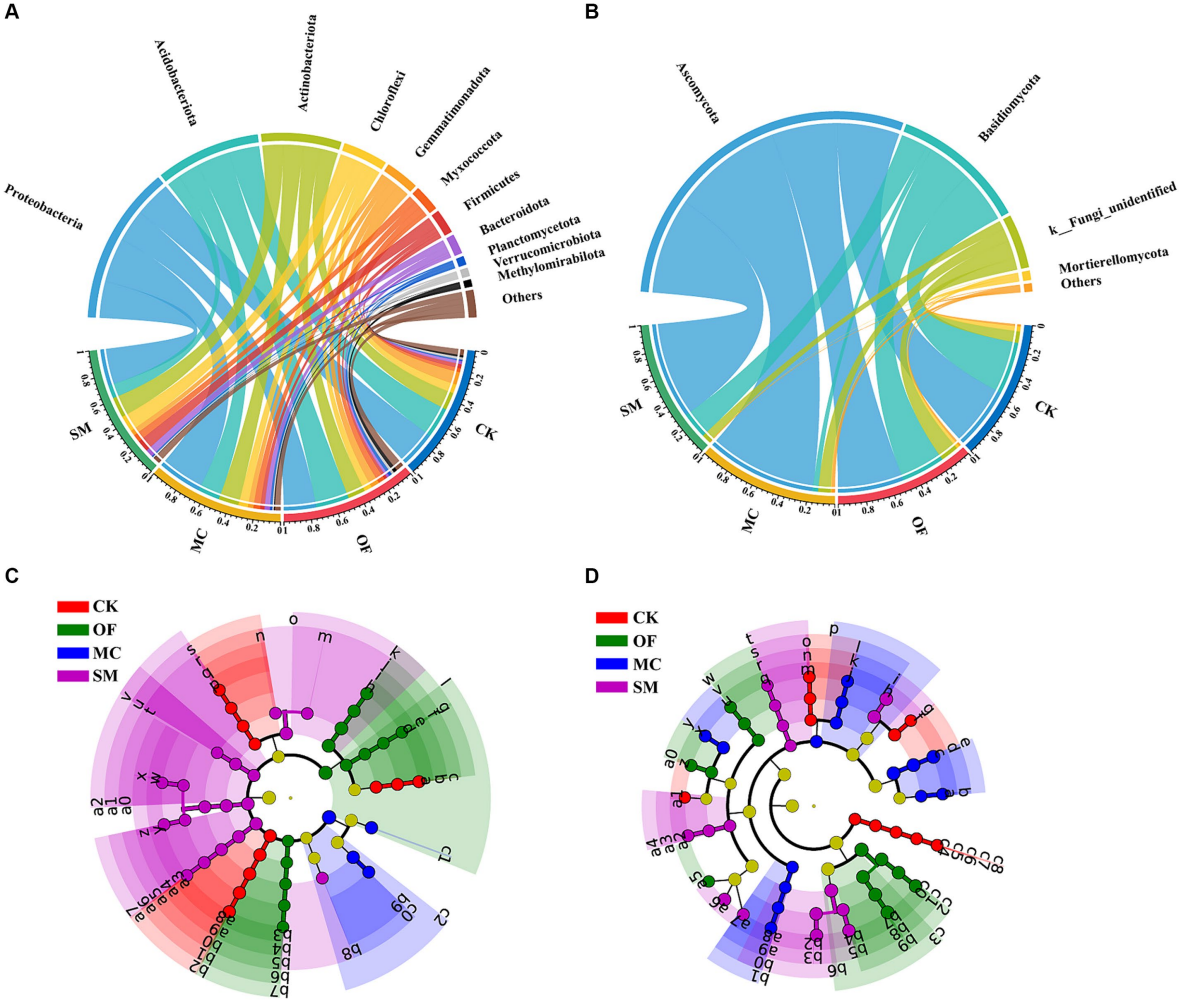
The differences in microbial community composition under RPBF treatments. Effects of different treatments on the relative abundance of communities at the phylum level for bacteria **(A)** and fungi **(B)**. The cladogram of bacterial **(C)** and fungal **(D)** biomarkers. The phyla with a relative abundance below 1% were grouped as “Others.” CK, no treatment; OF, application of organic fertilizer; MC, mushroom cultivation; SM, surface mulching.

We further analyzed the impact of different treatments on the community composition of bacteria and fungi. Among all samples, Proteobacteria, Acidobacteriota, Actinobacteriota, Chloroflexi, Gemmatimonadota, and Myxococcota were the dominant bacterial phyla, accounting for 82.34% of the bacterial community (Figure 4A). The OF treatment significantly increased the relative abundance of Planctomycetota and Methylomirabilota (*p* < 0.05; Supplementary Figures S1i,k), while the MC treatment led to a notable increase in Proteobacteria and Bacteroidota (*p* < 0.05; Supplementary Figures S1a,h). The SM treatment significantly augmented the relative abundance of Chloroflexi and Firmicutes (*p* < 0.05; Supplementary Figures S1d,g), but decreased the relative abundance of Acidobacteriota (*p* < 0.05; Supplementary Figure S1b). Compared to CK, all RPBF treatments significantly enhanced the relative abundance of Myxococcota but reduced the relative abundance of Gemmatimonadota (*p* < 0.05; Supplementary Figures S1e,f). In the fungal community, Ascomycota and Basidiomycota were the dominant fungal phyla, representing 87.3% of the fungal community (Figure 4B). The MC treatment significantly increased the relative abundance of Ascomycota (*p* < 0.05; Supplementary Figure S1l), but decreased the relative abundance of Basidiomycota (*p* < 0.05; Supplementary Figure S1m). Compared to CK, all RPBF treatments led to a significant decrease in Mortierellomycota (*p* < 0.05; Supplementary Figure S1o).

The LEfSe analysis further indicated that three RPBF treatments shaped distinct microbial communities (Figures 4C,D). It identified 49 bacterial and 55 fungal biomarkers across all treatments, with the SM treatment showing the highest number of bacterial biomarkers (19) and fungal biomarkers (16). Interestingly, the MC treatment exhibited more fungal (15) than bacterial biomarkers (4). Specifically, p_Gemmatimonadota, p_Mortierellomycota, o_Gaiellales, o_Thelebolales, g_Subgroup_2, g_Penicillium, and g_Fusarium were obviously enriched in the CK treatment (Supplementary Figure S2). Moreover, p_Acidobacteriota, p_Methylomirabilota, c_Vicinamibacteria, c_Tremellomycetes, o_Filobasidiales, f_Pyrinomonadaceae, g_RB41, g_Rokubacteriales, g_Coniochaeta, and g_Humicola were more enriched in the OF treatment. In the MC treatment, a notable enrichment of several microbial taxa was observed, such as p_Proteobacteria, c_Leotiomycetes, o_Rhizobiales, o_Burkholderiales, o_Tubeufiales, f_Hypocreaceae, g_Monocillium, and g_Leptosphaeria, p_Firmicutes, p_Chloroflexi, c_Actinobacteria. Lastly, the SM treatment was characterized by a significant increase in the abundance of c_Pezizomycetes, o_Polyangiales, o_Cytophagales, o_Agaricales, f_Stephanosporaceae, g_Bacillus, g_A4b, g_Thermomyces, and g_Mycothermus.

### 3.5 Effects of RPBF on microbial community function

**Figure 5:**
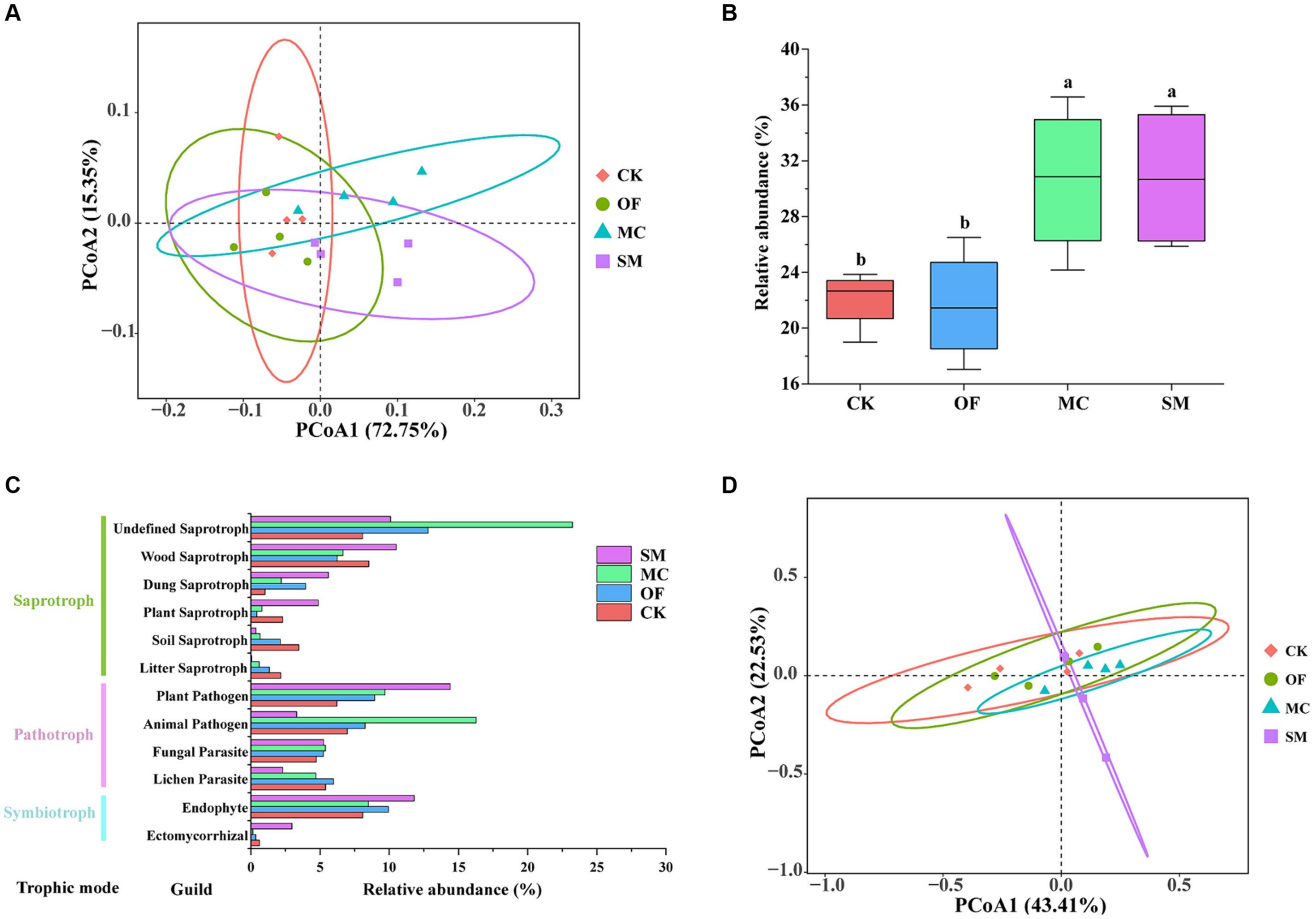
Soil bacterial function prediction by FAPROTAX and fungal function prediction by FUNGuild. Principal coordinate analysis (PCoA) of bacterial **(A)** and fungal **(D)** functions under different treatments. Effects of different treatments on the functions related to carbon cycle **(B)**. The fungal functional group profile **(C)**. CK, no treatment; OF, application of organic fertilizer; MC, mushroom cultivation; SM, surface mulching. The different letters in the figure indicate a significant difference at *p* < 0.05.

We utilized the FAPROTAX database to annotate bacteria and the result indicated 73 groups were represented. The bacterial functional Shannon index was significantly higher in the MC and SM treatments compared to the OF treatment (*p* < 0.05; Supplementary Figure S3a). Additionally, a clear distinction in bacterial functional communities among the different treatments was observed (PERMANOVA: *R*^2^ = 0.561, *p* = 0.004; Figure 5A). In comparison to CK, both MC and SM treatments significantly enhanced functions associated with the carbon cycle (Figure 5B). Specifically, the OF and MC treatments increased the relative abundance of photoautotrophy by 14.91 and 38.32%, and of anoxygenic photoautotrophy by 36.41 and 67.22%, respectively. Conversely, the SM treatment led to a decrease in both photoautotrophy and anoxygenic photoautotrophy by 30.63 and 29.40% (Supplementary Figures S4i,j). Futhermore, the MC and SM treatments significantly augmented the relative abundance of chemoheterotrophy, fermentation, and xylanolysis (*p* < 0.05; Supplementary Figures S4a,c,k). All RPBF treatments resulted in a significant decrease in (*p* < 0.05) cyanobacteria and oxygenic photoautotrophy (Supplementary Figures S4n,o). The SM treatment showed a significant increase (*p* < 0.05) in fumarate respiration and methanotrophy, compared to the other treatments (Supplementary Figures S4l,p).

For fungal communities, we utilized the FUNGuild database to infer their potential functions, identifying three primary trophic modes: saprotroph, pathotroth, and symbiotroph. Our results indicated that there are a total of 12 major functional group guilds (with a relative abundance of >1%; Figure 5C). The Shannon index of fungal function showed no significant differences across different treatments (*p* > 0.05; Supplementary Figure S3b). However, a distinct variation in fungal functional communities among treatments was observed (PERMANOVA: *R*^2^ = 0.331, *p* = 0.037; Figure 5D). In addition, the MC treatment significantly increased the relative abundance of undefined saprotroph and animal pathogen (*p* < 0.05; Supplementary Figures S5a,b). The relative abundance of dung saprotroph, plant saprotroph, and ectomycorrhizal was the highest in the SM treatment (Supplementary Figures S5d,e,h). All RPBF treatments significantly decreased the relative abundance of soil saprotroph and litter saprotroph (*p* < 0.05; Supplementary Figures S5f,g).

### 3.6 Co-occurrence network analysis of soil microbial communities

**Figure 6:**
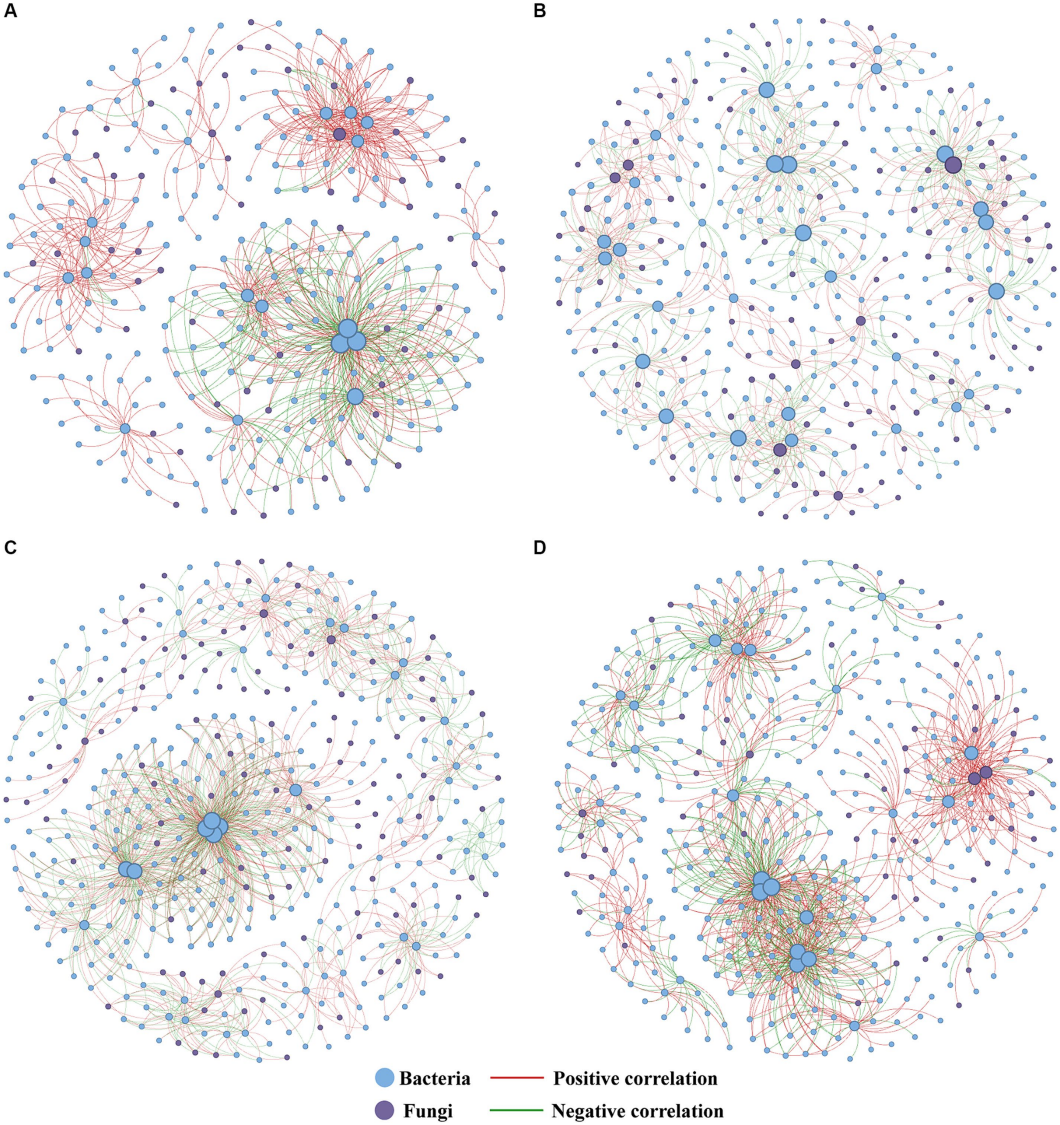
Co-occurrence network analysis of soil microorganisms under different CK **(A)**, OF **(B)**, MC **(C)**, and SM **(D)** treatments. The size of each node represents the degree of the node. CK, no treatment; OF, application of organic fertilizer; MC, mushroom cultivation; SM, surface mulching.

We further applied co-occurrence network analysis to reveal the general impact of different treatments on soil microbial communities (Figure 6) The results indicated that all RPBF treatments increased the number of nodes and edges. Moreover, there was a rise in the proportion of negative links, coupled with a reduction in the proportion of positive links. The network density in the MC treatment was the highest. In comparison to CK, the OF and MC treatments increased the number of fungal nodes and decreased the number of bacterial nodes, whereas the SM treatment exhibited the opposite trend (Supplementary Table S4).

### 3.7 Relationship between microbial communities and environmental factors

**Figure 7:**
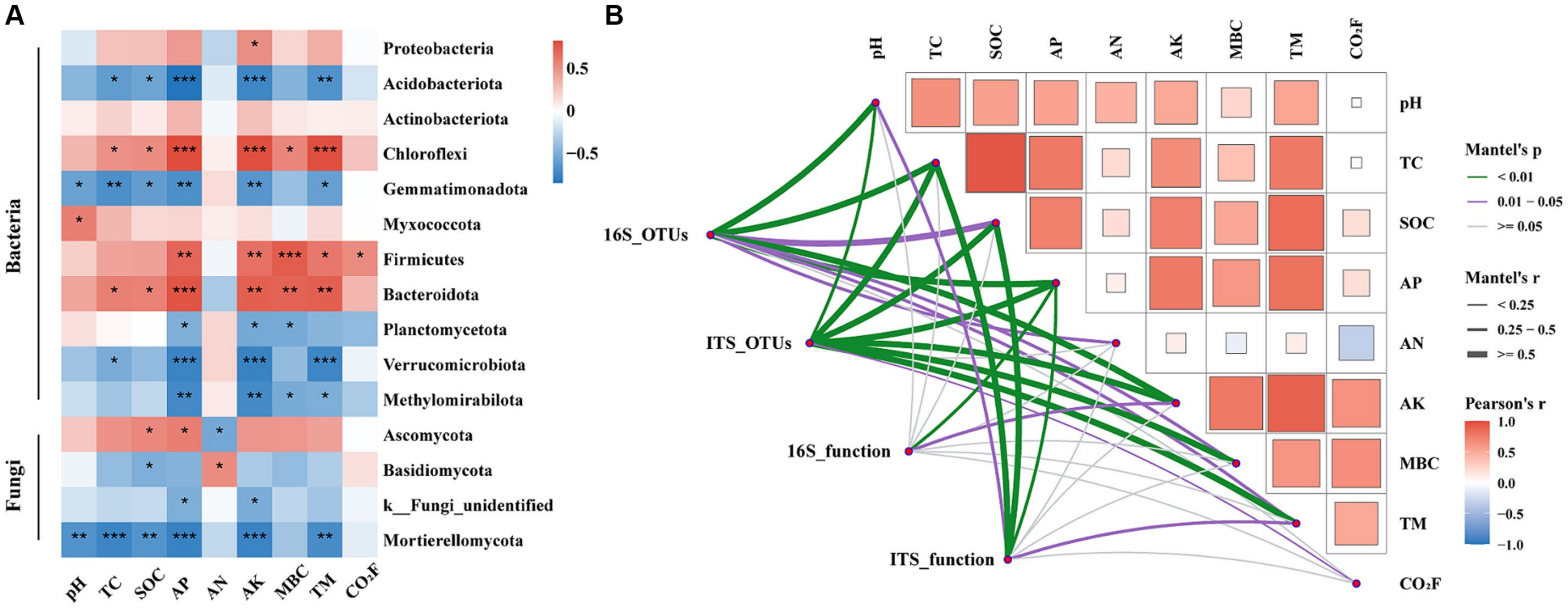
Spearman Correlation analysis of soil microorganisms on phylum level (with a relative abundance of >1%; **A**). The Mantel test showed correlations between the composition and function of bacterial and fungal communities and environmental factors **(B)**. AN, available nitrogen; AP, available phosphorus; AK, available potassium; TC, total carbon; SOC, soil organic carbon; MBC, microbial biomass carbon; TM, temperature; CO_2_F, carbon dioxide fluxes. In the figure, * indicates a significant difference at *p* < 0.05, ** indicates a significant difference at *p* < 0.01, *** indicates a significant difference at *p* < 0.001.

The correlation analysis revealed significant associations between the phylum level microbial communities and environmental factors (Figure 7A). Specifically, soil pH showed a positive correlation with Myxococcota and a negative correlation with Gemmatimonadota (*p* < 0.05). Both TC and SOC were significantly positively correlated with Chloroflexi and Bacteroidota, but negatively correlated with Acidobacteriota (*p* < 0.05). AP and AK exhibited significant negative correlations with Verrucomicrobiota and Mortierellomycota (*p* < 0.001). AN was significantly positively correlated with Basidiomycota (*p* < 0.05), while MBC was negatively correlated with Planctomycetota and Methylomirabilota (*p* < 0.05). TM and CO_2_ fluxes showed positive correlations with Firmicutes (*p* < 0.05). The Mantel test results further substantiated the microbial community composition and function were significantly correlated with environmental factors (Figure 7B). Soil pH was the strongest influence on bacterial community composition (Mantel’s *r* = 0.62, *p* < 0.01), while AK shows the strongest effect on fungal community composition (Mantel’s *r* = 0.65, *p* < 0.01). Among soil nutrients, AP had the strongest impact on microbial community function. Compared to bacteria, the composition (Mantel’s *r* = 0.40, 0.05 < *p* < 0.01 vs. Mantel’s *r* = 0.51, *p* < 0.01) and function (Mantel’s *r* = 0.01, *p* > 0.05 vs. Mantel’s *r* = 0.51, *p* < 0.01) of fungal communities demonstrated a stronger correlation with SOC.

**Figure 8:**
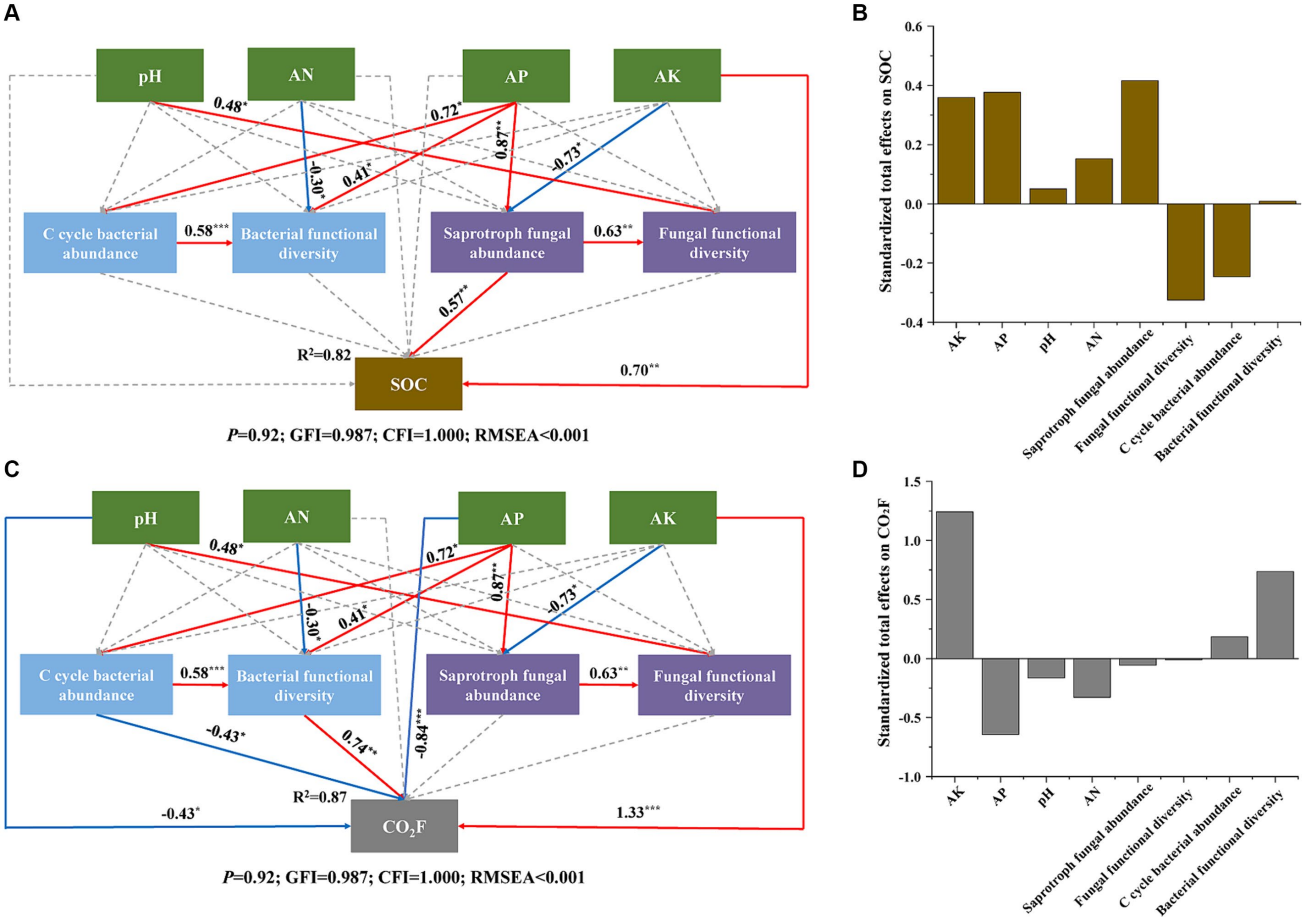
Structure equation model (SEM) of direct and indirect effects of soil abiotic and biotic properties on SOC **(A)** and CO_2_F **(C)**. Standardized total effects of these factors on SOC **(B)** and CO_2_F **(D)**. For C cycle bacterial abundance, we used the abundance of soil bacterial function related to the carbon cycle according to the function prediction by FAPROTAX. For saprotroph fungal abundance we used the abundance of saprophytic fungi according to the function prediction by FUNGuild. For the functional diversity of bacteria and fungi, we used their Shannon indices. Solid arrows indicate significant paths at *p < 0.05, **p < 0.01, ***p < 0.001, while dashed arrows indicate non-significant paths. The red and blue arrows represent positive effects and negative effects, respectively.

Our SEM model exhibited a reasonable fit, explaining 82% of the variation in SOC and 87% of the variation in CO_2_ fluxes (Figures 8A,C). The model revealed that AK and saprotroph fungal abundance directly influenced SOC, while AP and AK directly affected saprotroph fungal abundance, thereby indirectly influencing SOC. Moreover, pH, AP, and AK directly influenced CO_2_ fluxes. The CO_2_ fluxes were directly controlled by the abundance of soil bacterial function related to the carbon cycle (C cycle bacterial abundance) and bacterial functional diversity, and those two factors were influenced by AP and AN. C cycle bacterial abundance directly impacted bacterial functional diversity. Furthermore, AK, AP, and saprotroph fungal abundance showed a positive total effect on SOC. Conversely, fungal functional diversity and C cycle bacterial abundance had a negative total effect on SOC (Figure 8B). In addition, AK and bacterial functional diversity had a positive total effect on CO_2_ fluxes, while AP had a negative total effect (Figure 8D).

## 4 Discussion

### 4.1 Effects of RPBF on environmental factors

In our study, we observed that all three RPBF treatments improved soil nutrient content, indicating the potential of peach branch waste as an effective component of returning field materials. Specifically, OF treatment increased soil TC and AN content (Supplementary Table S2). This finding is consistent with Liu J. et al. (2021), who reported that organic fertilization elevates soil total organic matter (TOM) and AN content, and Hu et al. (2022), who observed an increase in soil TC content following organic fertilization. This suggests that the decomposition of organic material from peach branches contributes to the nutrient cycling essential for soil fertility. Additionally, MC treatment enhanced soil TC, AP, and AK content, which are similar to the results of previous studies (Gong et al., 2018; Yang et al., 2020), may be due to residual cultivation substrate providing nutrients to the soil. Consistently, SM treatment also resulted in increased soil TC and available nutrient content. Some studies showed that straw mulching could effectively increase soil nutrient content (Yin et al., 2022; Li et al., 2023). It indicates that peach branches, like straw, can serve as efficient mulching materials, likely due to their contribution to moisture retention and nutrient enrichment. Moreover, soil acidification was harmful to soil microbial activity and plant growth (Bolan et al., 2023), while we found that OF and MC treatments remarkably increased soil pH, keeping it within a suitable range for peach tree growth. Overall, this is consistent with our hypothesis (1) that RPBF treatment improves soil properties and thus provides favorable growing conditions for peach trees, which is essential for the yield and quality of peach fruits.

Notably, compared to CK, SOC content was significantly higher, and average CO_2_ fluxes were lower in the OF and MC treatments (Figure 1D). This suggests that these treatments may have a higher carbon use efficiency (CUE) of soil microorganisms, with more carbon use for biosynthesis rather than for respiration. Interestingly, we found a decrease in the MBC content in the OF and MC treatments, which could be due to its transformation into necromass (Liang et al., 2017, 2019; Tao et al., 2023). In contrast, SM treatment not only elevated SOC and MBC content but also increased average CO_2_ fluxes. This latter effect was likely attributable to a rise in average temperature (Figure 1C). As Wang M. et al. (2022) showed that the soil temperature of topsoil (0–30 cm) was easily increased by surface air temperature warming, which in turn enhanced microbial respiration (Nottingham et al., 2020; Yan et al., 2022). Furthermore, an increase in MBC under warming also could stimulate microbial respiration (Ma et al., 2018; Yang et al., 2023). Therefore, the warming effect of the coarse materials used for the SM treatment may have enhanced microbial respiration, resulting in increased CO_2_ emissions. In addition, warming exacerbates the positive priming effect in soil, accelerating the decomposition of native SOC following exogenous plant carbon inputs (Tao et al., 2024). This process may also be responsible for the increased CO_2_ emissions from the SM treatment. Generally, the above results are in line with our hypothesis 2 that RPBF promotes organic carbon accumulation, but the three RPBF treatments had different effects on CO_2_ emissions. The OF and MC treatments decreased CO_2_ emissions, while the SM treatment increased CO_2_ emissions, which may be related to changes in soil microbes.

### 4.2 Effects of RPBF on soil microbial community

We found that the OF and MC treatments changed microbial diversity, which was similar to the results of previous studies (Zhang et al., 2019; Song et al., 2021). One research was shown that microbial diversity was positively correlated with microbial CUE (Domeignoz-Horta et al., 2020). Notably, the microbial respiration: microbial growth relationship had a strong effect on microbial CUE (Domeignoz-Horta et al., 2020; Schnecker et al., 2023), whereas increased microbial diversity decreased soil respiration (Bastida et al., 2021), thereby may increase microbial CUE. Therefore, higher microbial diversity (bacterial Chao1 and Shannon index; fungal Chao1 index) in our study may be responsible for the potential increase in microbial CUE, thus promoting the accumulation of SOC. Additionally, in the face of warming temperatures, a greater microbial diversity is needed to cope with it to maintain microbial growth and ecosystem function (García et al., 2018). However, unlike OF and MC treatments, the SM treatment did not affect microbial diversity, consistent with the research results of straw mulching (Liu G. et al., 2021; Liu B. et al., 2023). Therefore, the warming effect of SM treatment may disrupt the stable relationship between microbial respiration and growth in the above situations, and consequently reducing microbial CUE, which is unfavorable for the mitigation of carbon emissions. This association underscores the potential of microbial diversity as a mediator of soil carbon transformation.

Additionally, our study is consistent with existing other orchard studies (Wang et al., 2020; Song et al., 2021; Liu C. et al., 2023), identifying that Proteobacteria, Acidobacteriota, Actinobacteriota, Chloroflexi, Gemmatimonadota, Ascomycota, and Basidiomycota were the dominant bacterial and fungal phyla in peach orchards soil. All three RPBF treatments significantly influenced the microbial community composition (Figures 3, 4A,B). Differences in soil microbial communities could drive ecological processes such as nutrient cycling and organic matter decomposition (Cheng et al., 2017; Kang et al., 2021). Specifically, the OF, MC, and SM treatments increased the relative abundance of Planctomycetota, Proteobacteria, and Firmicutes, respectively (Supplementary Figures S1a,g,i). Moreover, both MC and SM treatments led to an increase in Bacteroidota (Supplementary Figure S1h). Most previous studies showed that these bacterial phyla play pivotal roles in driving the soil carbon cycle (López-Mondéjar et al., 2016; Ren et al., 2018; Hu et al., 2022). Furthermore, Ascomycota, and Basidiomycota as the main decomposers, could decompose organic matter in the soil (Zeng et al., 2020). OF treatment increased the relative abundance of Ascomycota, and decreased Basidiomycota, possibly due to their competition with each other (Supplementary Figures S1l,m). Our findings also reveal that RPBF treatments not only regulate the carbon cycle and organic matter decomposition but also influence the balance between plant pathogenic and beneficial microorganisms. The enrichment of beneficial microbes such as *Solicoccozyma*, *Humicola*, Rhizobiales, and *Bacillus* in three RPBF treatments highlights the potential of the RPBF treatment to promote plant growth through mechanisms like bioremediation, disease resistance, nitrogen fixation, and phytohormones secretion (Supplementary Figure S2; Yang et al., 2014; Erlacher et al., 2015; Aloo et al., 2019; Stosiek et al., 2019). However, *Fusarium* and *Penicillium* were plant pathogens that are harmful to plant health (Thambugala et al., 2020; Ren et al., 2024), which were enriched in CK treatment. These results suggest that RPBF treatment regulates carbon cycling and promotes plant growth by altering the composition of the microbial community.

In addition, we explored the effects of three RPBF treatments on bacterial community function, especially the functions related to the carbon cycle (Figures 5A,B). The OF and MC treatments increased the relative abundance of photoautotrophy and anoxygenic photoautotrophy, indicating an enhanced capacity for CO_2_ fixation into SOC, which in turn leads to the accumulation of SOC and reduction of CO_2_ emissions (Supplementary Figures S4i,j; Wu et al., 2024). The improvement of pH may be a key factor in increasing the abundance of autotrophic microorganisms (Supplementary Table S2; Huang et al., 2018; Hu et al., 2024). Furthermore, we found that soil pH had a positive total effect on SOC content and a negative total effect on CO_2_ fluxes (Figures 8C,D). This also suggests that soil pH has a crucial role in soil carbon accumulation (Malik et al., 2018). However, the alkaline soil pH may be unfavorable for the growth of autotrophic microorganisms, which results in SM treatment decreasing the relative abundance of photoautotrophy and anoxygenic photoautotrophy. In addition, SM treatment also increased the relative abundance of chemoheterotrophy and fermentation (Supplementary Figures S4a,c; Yu et al., 2021; Liu B. et al., 2023). These results indicate a potential additional reason why SM treatment led to an increase in CO_2_ emissions. Moreover, three RPBF treatments significantly affected fungal community function (Figures 5C,D). The relative abundance of soil saprotroph was reduced in all RPBF treatments (Supplementary Figure S5f), which might be attributed to the RPBF treatment likely providing sufficient nutrients, diminishing the soil ecosystem’s reliance on the soil saprotroph for decomposition of organic materials, such as plant residues and necromass, for nutrient provision. Consequently, this process is conducive to the accumulation of SOC. Notably, SM treatment exhibited the highest relative abundance of xylanoysis and plant saprotroph (Supplementary Figures S4k, S5e). This is likely because peach branches contain rich amounts of lignin, cellulose, and hemicellulose, thus stimulating the growth of specific functional microbial communities (Guo et al., 2021; Wei et al., 2023). Collectively, the three RPBF treatments shaped distinct microbial community functions, which in turn regulate SOC content and CO_2_ fluxes.

Among three RPBF treatments, the SM network was observed to have a higher proportion of positive links, potentially indicative of enhanced cooperative interactions within the network during the decomposition of peach branches (Figure 6D and Supplementary Table S4). It was consistent with the research results of straw mulching (Li et al., 2023). In contrast, the MC network displayed a greater proportion of negative links, likely due to the introduction of mushrooms as exogenous microorganisms influencing the local microbial communities (Figure 6C and Supplementary Table S4; Sun et al., 2020). Compared to the CK, all RPBF treatments increased the proportion of negative links (Figure 6 and Supplementary Table S4). This may be attributed to the potential for more competition and antagonism to lead to more negative interactions between microorganisms in high nutrient concentration environments, while microorganisms tend to coexist in environments with low nutrient concentrations (Ratzke et al., 2020; Liu J. et al., 2021; Liu C. et al., 2023). Furthermore, the facilitative interactions between indigenous microorganisms promote pathogen invasions, while antagonistic interactions might play a crucial role in pathogen suppression (Li et al., 2019). This implies that the microbial networks within RPBF treatments might exhibit stronger resistance to pathogens due to their increased proportion of negative links.

### 4.3 Effects of RPBF on soil carbon cycle

AP and AK are important factors that alter the composition and function of soil microbial communities (Hu et al., 2022; Yin et al., 2022). In our study, both Spearman correlation analysis and the Mantel test revealed a significant association between AP and AK with the composition and function of microbial communities (Figure 7). Furthermore, our SEM further highlighted that AP and AK regulate SOC content and CO_2_ fluxes by influencing microbial communities. (Figures 8A,B). These findings emphasize the pivotal role of AP and AK in regulating soil carbon cycling within peach orchard ecosystems.

Notably, the contribution of microbial communities to soil carbon sequestration is different, communities dominated by fungi have stronger carbon sequestration capabilities, compared with communities dominated by bacteria (Six et al., 2006; Malik et al., 2016). Our analysis indicates that fungal communities, especially the abundance of saprophytic fungi, have a more positive total effect on SOC content than bacterial communities. Conversely, bacterial communities, especially bacterial functional diversity, have a more positive total effect on CO_2_ fluxes (Figures 8B–D). This suggests that in peach orchards, fungal communities contribute more to carbon sequestration, while bacterial communities are more influential in carbon emission. Additionally, OF and MC treatment increased the number of fungal nodes in their network, while SM treatment increased the number of bacterial nodes (Figure 6D and Supplementary Table S4). This may be caused by bacteria having competitive advantages under warming (Hu et al., 2023). Consequently, the observed increase in CO_2_ fluxes with SM treatment and the decrease following OF and MC treatments may be attributed to the enhanced or diminished presence of bacterial communities within the soil microbial communities.

Overall, environmental factors regulate the soil carbon cycle by influencing microbial communities, thereby confirming the mediating role of microbial communities and supporting our hypothesis. In the future, we aim to evaluate the long-term effects of RPBF treatments on the ecological and economic benefits of peach orchards. In addition, we plan to adopt metagenomic methods to further reveal the impact of soil microbes on the dynamic changes of soil carbon. Meanwhile, more suitable methods for returning peach branch waste to fields will be screened during long-term field management practices.

## 5 Conclusion

This study systematically evaluated the effects of three different RPBF treatments on soil chemical property, microbial community, and carbon cycling in a peach orchard. All RPBF treatments effectively improved soil nutrient content and modified the soil microbial community composition by increasing beneficial microorganisms while suppressing harmful ones. Consequently, the RPBF treatment created a more conducive environment for peach tree growth. Meanwhile, the improved environment enhanced the potential for soil carbon sequestration and emission reduction. The increase in the abundance of autotrophic microorganisms leads to greater storage of SOC. Additionally, AP and AK, as key factors, affect soil carbon cycling by influencing microbial community composition and function. In peach orchard soil, fungal communities were found to contribute more greatly to SOC content than bacterial communities. However, SM treatment increased the presence of bacteria in the microbial community due to warming and also diminished their carbon fixation function, resulting in increased CO_2_ emissions. In contrast, OF and MC treatments increased the presence of fungi in the microbial community and also enhanced the carbon fixation function of the bacterial community. Therefore, OF and MC treatments emerged as more effective for promoting soil carbon sequestration and emission reduction. These findings provide new perspectives for the scientific resource utilization of peach branch waste, contributing towards the sustainable development of green agriculture.

## Data availability statement

The datasets presented in this study can be found in online repositories. The names of the repository/repositories and accession number(s) can be found at: https://www.ncbi.nlm.nih.gov/, PRJNA1070042 for bacteria and PRJNA1070043 for fungi.

## Author contributions

CL: Conceptualization, Data curation, Software, Visualization, Writing – Original Draft. ZL: Conceptualization, Formal analysis, Writing – Original Draft. BC: Investigation, Software, Writing – Original Draft. HY: Supervision, Investigation, Writing – Original Draft. CG: Investigation, Funding acquisition, Writing – Original Draft. MC: Conceptualization, Writing – review & editing. YL: Conceptualization, Supervision, Writing – review & editing.
