# Supplementary material for: Effects of returning peach branch waste to fields on soil carbon cycle mediated by soil microbial communities
Source: Front Microbiol. 2024 Jun 18;15:1406661. doi: 10.3389/fmicb.2024.1406661 (PMC11217190; doi:10.3389/fmicb.2024.1406661)
Supplement: Supplementary file 1 [file Data_Sheet_1.docx]

Supplementary Material

Effects of returning peach branch waste to fields on soil carbon cycle mediated by soil microbial communities

# Supplementary Figures and Tables

## Supplementary Figures


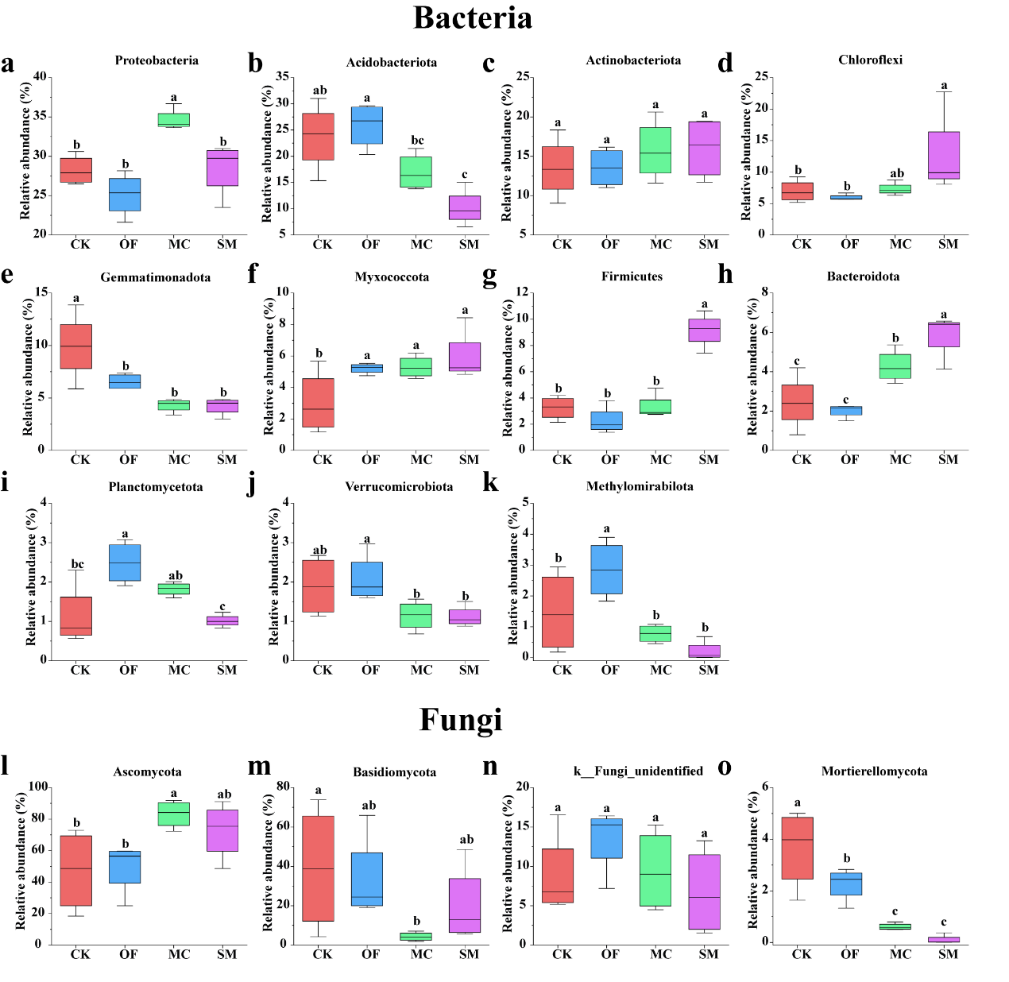


**Supplementary Figure 1.** The significant differences in the relative abundance of the top 1% phyla bacteria (a-k) and fungi (l-o) between different treatments.


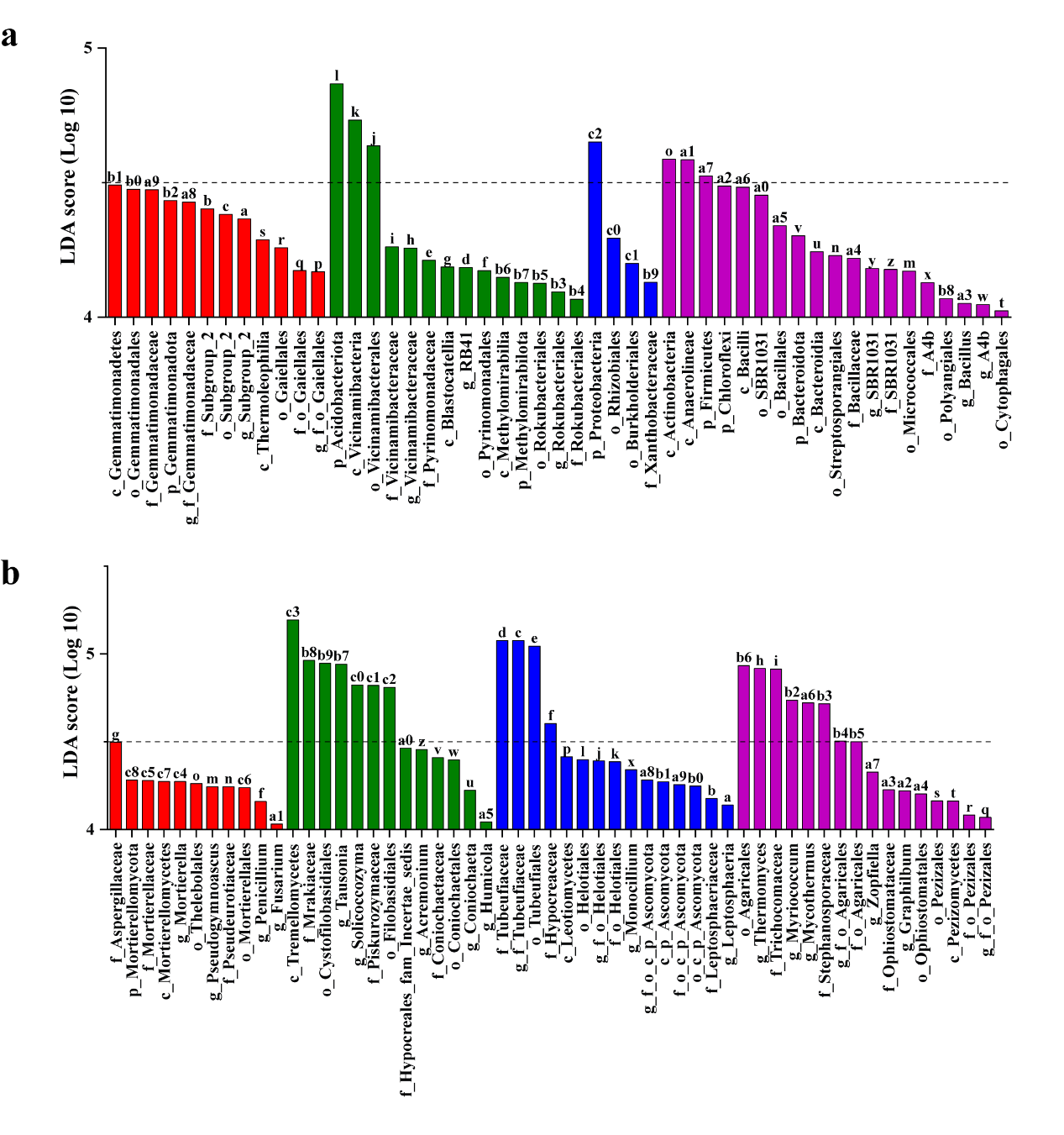


**Supplementary Figure 2.** The histogram of LDA scores for bacteria (a) and fungi (b).


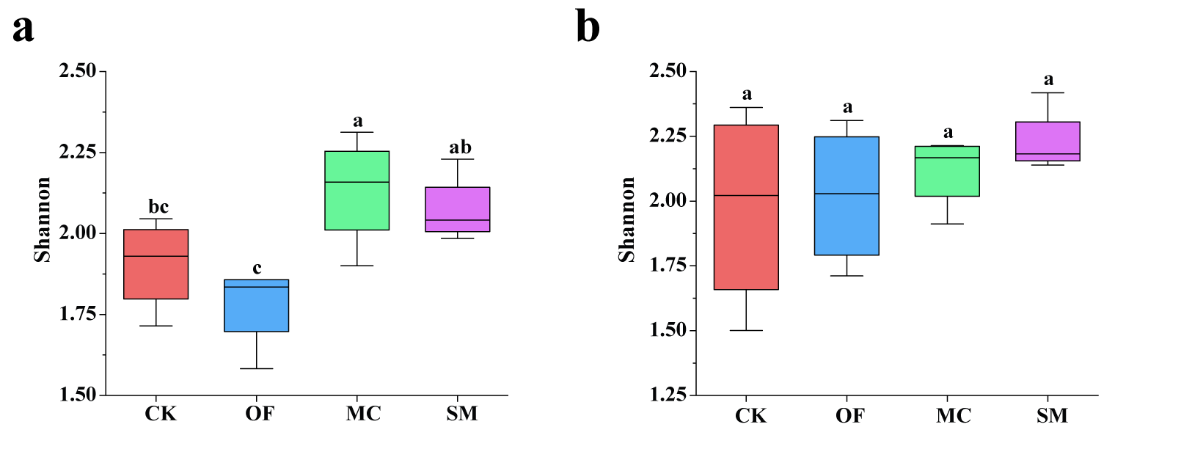


**Supplementary Figure 3.** The significant differences in the Shannon index of bacterial function (a) and fungal function (b) between different treatments.


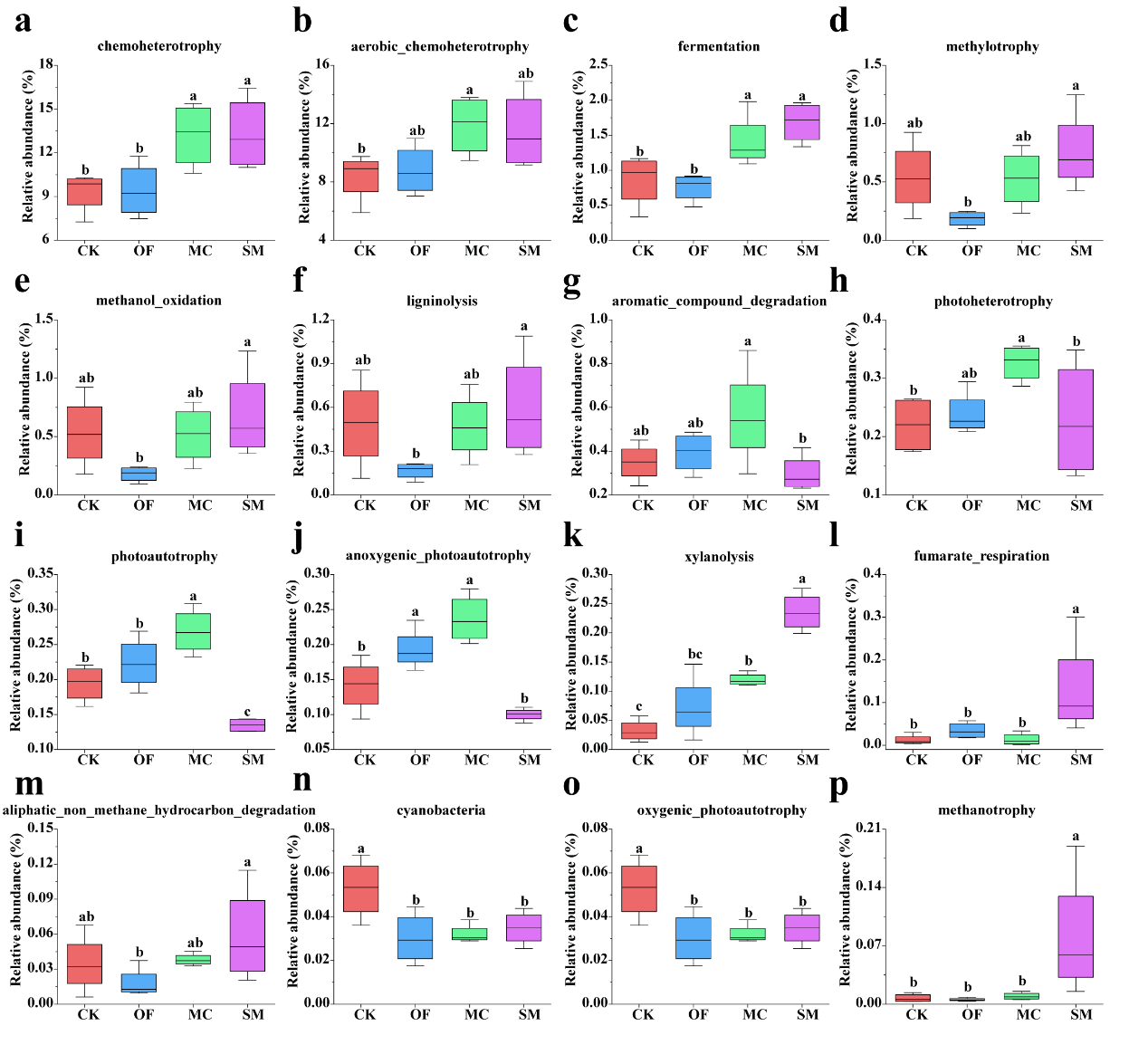


**Supplementary Figure 4.** The significant differences in relative abundance of bacterial functions between different treatments.


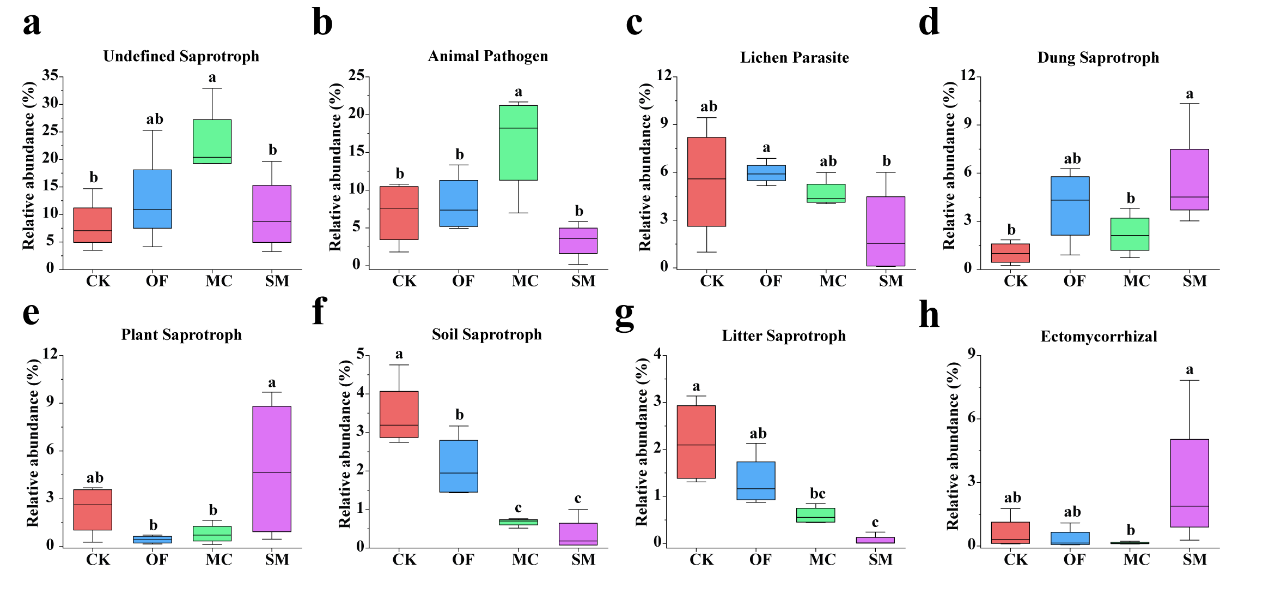


**Supplementary Figure 5.** The significant differences in relative abundance of fungal functions between different treatments.

## Supplementary Tables

**Supplementary Table 1.** Chemical properties of soil and three RPBF treatments before the experiment.

| **Chemical properties** | **Soil** | **OF** | **MC** | **SM** |
| --- | --- | --- | --- | --- |
| pH | 6.68 | 7.38 | 7.73 | 7.33 |
| TC（g/kg） | 11.30 | 119.75 | 295.00 | 335.00 |
| SOC（g/kg） | 10.05 | 106.25 | 265.00 | 312.00 |
| AN（mg/kg） | 13.00 | 209.00 | 338.33 | 662.00 |
| AP（mg/kg） | 100.27 | 304.25 | 150.67 | 276.75 |
| AK（mg/kg） | 477.75 | 2887.50 | 6572.50 | 11127.50 |

**Supplementary Table 2.** Soil chemical properties under RPBF treatments.

| **Treatment** | **pH** | **TC（g/kg）** | **SOC（g/kg）** | **AN（mg/kg）** | **AP（mg/kg）** | **AK（mg/kg）** | **MBC（µg/g）** |
| --- | --- | --- | --- | --- | --- | --- | --- |
| CK | 6.68 ± 0.43b | 11.30 ± 2.02c | 10.05 ± 2.01b | 13.00 ± 4.14a | 100.27 ± 22.54c | 447.75 ± 59.69bc | 449.43 ± 138.55b |
| OF | 7.63 ± 0.27a | 21.65 ± 2.57bc | 15.10 ± 1.56b | 19.67 ± 0.62a | 58.98 ± 6.51c | 294.00 ± 28.43c | 114.90 ± 52.58b |
| MC | 7.60 ± 0.10a | 36.40 ± 3.81ab | 24.93 ± 1.60b | 15.33 ± 0.24a | 194.50 ± 16.92b | 685.50 ± 39.07b | 328.33 ± 152.36b |
| SM | 8.20 ± 0.23a | 49.53 ± 10.70a | 43.47 ± 10.68a | 18.00 ± 1.47a | 247.67 ± 15.15a | 2542.50 ± 145.74a | 949.40 ± 74.09a |

**Supplementary Table 3.** Pairwise Adonis test of PCoA results.

| **Pairwise adonis test** |  | **R^2^** | ***p*** |
| --- | --- | --- | --- |
| Bacteria | CK vs OF | 0.328 | 0.064 |
|  | CK vs MC | 0.356 | 0.034 |
|  | CK vs SM | 0.392 | 0.035 |
|  | OF vs MC | 0.459 | 0.032 |
|  | OF vs SM | 0.530 | 0.028 |
|  | MC vs SM | 0.431 | 0.023 |
| Fungi | CK vs OF | 0.321 | 0.032 |
|  | CK vs MC | 0.308 | 0.034 |
|  | CK vs SM | 0.376 | 0.035 |
|  | OF vs MC | 0.430 | 0.032 |
|  | OF vs SM | 0.454 | 0.028 |
|  | MC vs SM | 0.416 | 0.023 |

**Supplementary Table 4.** Co-occurrence network topological properties of soil microbial communities in peach orchards under different treatments.

| **Topological properties** | **CK** | **OF** | **MC** | **SM** |
| --- | --- | --- | --- | --- |
| Number of nodes | 293 | 441 | 452 | 431 |
| Number of edges | 830 | 971 | 1424 | 1205 |
| Average degree | 5.67 | 4.40 | 6.30 | 5.59 |
| Positive links (%) | 73.61% | 56.54% | 54.14% | 63.82% |
| Negative links (%) | 26.39% | 43.46% | 45.86% | 36.18% |
| Network density | 0.019 | 0.010 | 0.020 | 0.013 |
| Modularity | 0.65 | 0.81 | 0.63 | 0.67 |
| Number of bacteria (%) | 79.86% | 75.74% | 73.67% | 86.31% |
| Number of fungi (%) | 20.14% | 24.26% | 26.33% | 13.69% |
